# Persistent facial erythema in an infant

**DOI:** 10.1016/j.jdcr.2025.01.038

**Published:** 2025-03-27

**Authors:** Sarah Lee, Fiona Lynch, Pierre-Olivier Grenier

**Affiliations:** a Harvard Medical School, Boston, Massachusetts; b Dermatology Section, Division of Immunology, Department of Pediatrics, Boston Children’s Hospital, Boston, Massachusetts

**Keywords:** infant, lupus, neonatal lupus erythematosus, pediatric dermatology

## History

**Figure 1:**
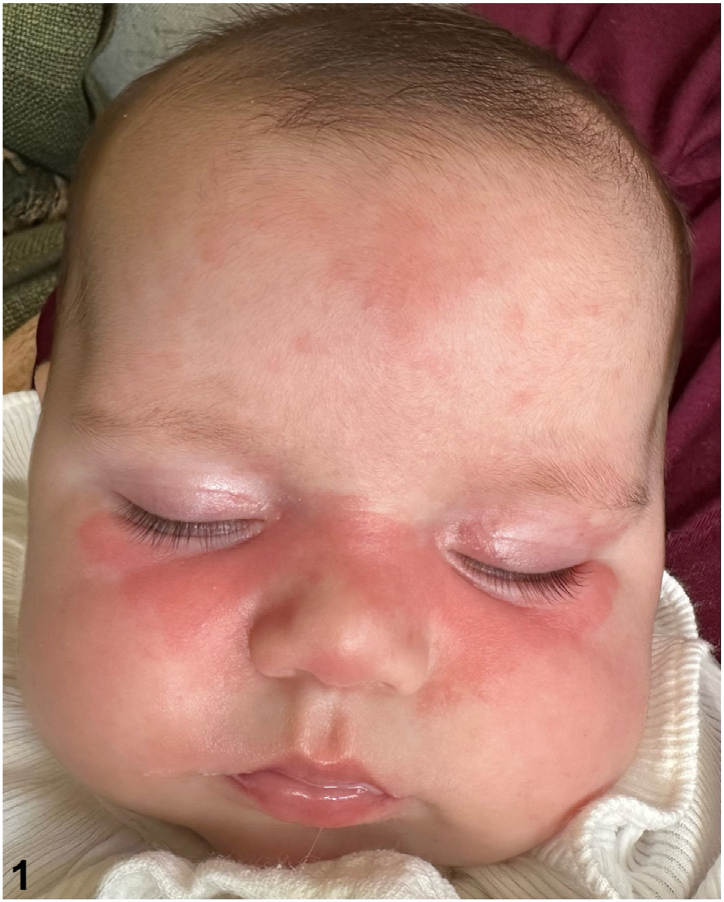

A 3-month-old female presented with a one-month history of a thin pink plaque involving the lower eyelids, nose, cheeks and upper forehead (Fig 1). This persisted despite regular moisturization and was exacerbated by sun exposure. There was no history of medication use or exogenous irritant exposure. Family history was notable for multiple sclerosis and Grave’s disease in the mother, psoriasis in the father, and atopic dermatitis in the sister. She was born at full-term by uncomplicated vaginal delivery. Antenatal maternal antibody screening was not performed. Postnatal weight, length, head circumference, cardiac examination, and rest of skin examination were normal. Laboratory testing was ordered.

**Question 1: Which of the following is the most likely diagnosis?**

A. Bloom syndrome
B. Neonatal lupus erythematosus (NLE)
C. Seborrheic dermatitis
D. Cutaneous phototoxicity
E. Rothmund-Thomson syndrome

**Answers:**

**A.** Bloom syndrome – Incorrect. Bloom syndrome is an autosomal recessive disorder that can manifest with sun-sensitive facial erythema. However, other features of the condition, including café-au-lait macules, failure to thrive, and immunodeficiency were lacking.^1^
**B.** NLE – Correct. Cutaneous NLE presents within the first few weeks of life, predominantly involving the periorbital (“raccoon eyes”) and malar areas.^2^ Although features such as annular plaques, papulosquamous lesions, hypo/hyperpigmentation, telangiectasias, or atrophy were lacking, the maternal history of autoimmunity, photosensitivity, and distribution favored cutaneous NLE. The diagnosis was confirmed by a positive Ro60 (SSA) antibody (Table I).

**Table I:** Results of laboratory testing

Laboratory test	Result	Normal Range	Interpretation
Anti-nuclear antibody	Detected, < 1:80	< 1:80	Weak positive
Ro52 (SSA) antibody, IgG	4 AU/mL	0 – 40	Negative
Ro60 (SSA) antibody, IgG	98 AU/mL	0 – 40	Positive
La (SSB) antibody, IgG	1 AU/mL	0 – 40	Negative
Hemoglobin	11.5 g/dL	9.6 – 13.2	Normal
White blood cell count	10.91 K cells/uL	6.8 – 14.63	Normal
Absolute neutrophil count	0.74 K cells/uL	1.37 – 5.68	Low
Platelet count	488 K cells/uL	286 – 579	Normal
ALT	46 unit/L	3 - 54	Normal
AST	35 unit/L	10 -65	Normal
**C.** Seborrheic dermatitis – Incorrect. Infantile seborrheic dermatitis features greasy, yellow scale, primarily on the scalp but also seen on the centrofacial area, including the central and lower forehead, eyebrows, and nasolabial folds. In this case, the absence of scale and scalp involvement, along with the notable presence of photosensitivity, was inconsistent with seborrheic dermatitis.
**D.** Cutaneous phototoxicity – Incorrect. Cutaneous phototoxicity results from UV-induced activation of a phototoxic compound in the skin, typically from a drug or plant (phytophotodermatitis), causing direct cellular injury.^3^ It presents with sunburn-like rash that resolves with peeling and hyperpigmentation.^3^ The absence of a history of such exposure and the prolonged course of symptoms were inconsistent with this diagnosis.
**E.** Rothmund-Thomson syndrome – Incorrect. Rothmund-Thomson syndrome, a rare genodermatosis, initially presents with erythema, edema, and blistering on the face, extremities, and buttocks, later progressing to poikiloderma. This patient lacked other features like sparse, brittle hair, skeletal abnormalities, and short stature.^1^

**Question 2: The diagnosis of NLE was confirmed when lab testing revealed a positive anti-Ro60 (SSA) antibody (98 AU/mL). What is the most important clinical investigation to order next?**

A. Liver function tests (LFTs)
B. Anti-Smith antibody
C. Electrocardiogram (EKG)
D. Skin biopsy
E. Complete blood count (CBC)

**Answers:**

**A.** LFTs – Incorrect. While LFTs should be ordered to screen for hepatic involvement (which may present as hepatomegaly, abnormal LFTs, or rarely, liver failure) the priority is to evaluate cardiac manifestations, as they are more common and severe.^2^ This patient’s LFTs were normal.
**B.** Anti-Smith antibody – Incorrect. Anti-Smith antibody, a highly specific marker of systemic lupus erythematosus, is not of diagnostic or prognostic utility in NLE.
**C.** EKG – Correct. An EKG should be performed as a priority to assess for congenital heart block. This potentially life-threatening manifestation of NLE occurs in 15-30% of infants and is mediated by maternal Anti-Ro (SSA).^2^ Reassuringly, our patient’s EKG showed normal sinus rhythm, without findings of atrioventricular block or arrythmia.
**D.** Skin biopsy – Incorrect. Skin biopsy is not a priority, as it is not necessary for diagnosis or management of NLE.
**E.** CBC – Incorrect. CBC should be performed to investigate for hematologic complications of NLE including leukopenia, anemia, and thrombocytopenia, but EKG is a more urgent investigation.

**Question 3: Which of the following statements is true regarding the prognosis of NLE?**

A. Infants with NLE have a 20% chance of developing systemic lupus erythematosus (SLE) in later life
B. The cutaneous lesions resolve spontaneously over the first 6 to 12 months of life
C. The risk of NLE in newborn sibling of an affected infant is negligible
D. Systemic therapies (such as glucocorticoids) can reverse complete heart block
E. Hydroxychloroquine taken during pregnancy has been found to reduce noncardiac manifestations of NLE

**Answers:**

**A.** Infants with NLE have a 20% chance of developing SLE in later life – Incorrect. NLE itself does not predispose affected infants to SLE later in life. Affected infants and their siblings do have a slightly elevated risk when compared to the general population, reflecting their genetic predisposition to autoimmunity.^4^ However, up to 50% of previously asymptomatic mothers of affected infants develop SLE or Sjogren’s syndrome later in life.^4^
**B.** The cutaneous lesions generally resolve spontaneously over the first 6 to 12 months of life – Correct. Clearance of skin findings occurs concurrently with the decline of maternal anti-Ro (SSA) antibodies in the infant’s circulation.^2^ While most lesions resolve without residual effects, some may leave telangiectasia, atrophy, or scarring. Sun protection is essential to prevent flares and minimize the risk of post-inflammatory dyspigmentation.
**C.** The risk of NLE in newborn sibling of an affected infant is negligible – Incorrect. Mothers with anti-Ro (SSA) or anti-La (SSB) antibodies have approximately a 36% risk of NLE recurrence in subsequent pregnancies, with a sixfold increased risk of cardiac manifestations compared to the first child.^4^ Hydroxychloroquine use in mothers can reduce this risk.^5^
**D.** Systemic therapies (such as glucocorticoids) can reverse complete heart block – Incorrect. Systemic therapies such as glucocorticoids and intravenous immunoglobulin are generally ineffective in reversing third-degree heart block, with 57-66% of infants requiring a pacemaker.^2^ Heart block results from anti-Ro (SSA) antibodies binding to fetal cardiac myocytes, damaging the conduction system.^2^
**E.** Hydroxychloroquine taken during pregnancy has been found to reduce noncardiac manifestations of NLE – Incorrect. Hydroxychloroquine reduces the risk of cardiac complications but does not prevent noncardiac manifestations.^5^

## Conflicts of interest

None disclosed.
